# Evaluation of breeding strategies for polledness in dairy cattle using a newly developed simulation framework for quantitative and Mendelian traits

**DOI:** 10.1186/s12711-016-0228-7

**Published:** 2016-06-29

**Authors:** Carsten Scheper, Monika Wensch-Dorendorf, Tong Yin, Holger Dressel, Herrmann Swalve, Sven König

**Affiliations:** Department of Animal Breeding, University of Kassel, 37213 Witzenhausen, Germany; Institute of Agricultural and Nutritional Sciences, University of Halle, 06099 Halle, Germany

## Abstract

**Background:**

Intensified selection of polled individuals has recently gained importance in predominantly horned dairy cattle breeds as an alternative to routine dehorning. The status quo of the current polled breeding pool of genetically-closely related artificial insemination sires with lower breeding values for performance traits raises questions regarding the effects of intensified selection based on this founder pool.

**Methods:**

We developed a stochastic simulation framework that combines the stochastic simulation software QMSim and a self-designed R program named QUALsim that acts as an external extension. Two traits were simulated in a dairy cattle population for 25 generations: one quantitative (QMSim) and one qualitative trait with Mendelian inheritance (i.e. polledness, QUALsim). The assignment scheme for qualitative trait genotypes initiated realistic initial breeding situations regarding allele frequencies, true breeding values for the quantitative trait and genetic relatedness. Intensified selection for polled cattle was achieved using an approach that weights estimated breeding values in the animal best linear unbiased prediction model for the quantitative trait depending on genotypes or phenotypes for the polled trait with a user-defined weighting factor.

**Results:**

Selection response for the polled trait was highest in the selection scheme based on genotypes. Selection based on phenotypes led to significantly lower allele frequencies for polled. The male selection path played a significantly greater role for a fast dissemination of polled alleles compared to female selection strategies. Fixation of the polled allele implies selection based on polled genotypes among males. In comparison to a base breeding scenario that does not take polledness into account, intensive selection for polled substantially reduced genetic gain for this quantitative trait after 25 generations. Reducing selection intensity for polled males while maintaining strong selection intensity among females, simultaneously decreased losses in genetic gain and achieved a final allele frequency of 0.93 for polled.

**Conclusions:**

A fast transition to a completely polled population through intensified selection for polled was in contradiction to the preservation of high genetic gain for the quantitative trait. Selection on male polled genotypes with moderate weighting, and selection on female polled phenotypes with high weighting, could be a suitable compromise regarding all important breeding aspects.

**Electronic supplementary material:**

The online version of this article (doi:10.1186/s12711-016-0228-7) contains supplementary material, which is available to authorized users.

## Background

The routinely-used practice of dehorning in dairy and beef cattle worldwide has attracted increased negative public perception, and contributes to conflicts between modern intensive livestock management and animal welfare. Undoubtedly, it is well documented that dehorning of calves is associated with stress, pain and temporary negative impact on calf growth [1–3]. Currently, the dehorning procedures used aim at improving animal welfare [4–6] by alleviating or even eliminating pain reactions which has led to the development of procedures regulated by legal prohibition [7]. Hence, the urgent need for alternatives to cattle dehorning is strengthened. Simply avoiding dehorning by keeping naturally-horned cattle is considered as one possibility [8], but implies substantial adaptation of housing conditions and does not contribute to reduce the risk of injuries among cattle and animal keepers [9, 10]. In this regard, targeted selection and dissemination of genetically-polled animals into naturally-horned cattle breeds (i.e. via introgression of polled alleles), appears to be the most practicable and sustainable solution.

Two loci, “*polled*” and “*scurs*”, determine the variety of phenotypes that are associated with the polled trait in cattle [11–18]. While the precise molecular structure and specific inheritance pattern of the *scurs* locus is not conclusively clarified, the *polled* locus in cattle has been mapped to the proximal end of bovine autosome 1 (BTA1, BTA for *Bos taurus*). The *polled* locus is characterized by (1) autosomal dominant inheritance of mutant alleles and (2) structural heterogeneity that depends on the origin of a breed. Two breed-specific haplotypes were identified at the *polled* locus. The Celtic allele present in Angus, Simmental, Limousin, Charolais, etc. is a complex insertion-deletion (indel), whereas breeds of Friesian origin (e.g. Holstein and Jersey) share a 80 kbp duplication as the most likely causative variant [11, 13, 14]. Since these variants are localized in non-coding DNA regions [15, 18], they are assumed to have rather a regulatory than directly a functional effect. Consequently, the identification of the molecular structure at the *polled* locus has led to the development of a validated direct gene test, which allows precise genotyping as required for substantiated selection decisions [19].

As reflected by the present number of entirely polled breeds and breeds with a significant ratio of polled individuals, breeding and selection for polled animals has a longer tradition in beef than in dairy cattle [20–22]. Thus, the most prevalent dairy cattle breeds in Europe (i.e. Holstein and Jersey) are characterized by a small proportion of polled animals, which is due to the initiative of a limited number of motivated “polled breeders” [20, 23]. It is only during the last decade that a slow but steadily increasing demand for polled artificial insemination (AI) sires has resulted in increasing the numbers of available polled Holstein AI bulls worldwide. Due to the limited number of polled founders, groups of polled Holstein individuals display lower average breeding values and a higher average kinship than horned individuals [21, 22, 24, 25]. These findings were recently confirmed by own evaluations based on the database from the German national genetic evaluation for Holstein AI sires [26]. Quite similar results were reported for dual-purpose German Simmental cattle, while no differences between polled and horned groups were found with regard to health, growth and reproductive traits in beef cattle [10, 27–29]. Evidence accumulated for polled German Simmental cattle, as well as more recent advances in the Holstein breed, further indicate that the initial inferior performance of polled individuals might be due to a selection advantage of their horned pendants rather than an inevitable genetic disadvantage [22, 30].

Based on these assumptions and comparisons between groups of horned and polled animals using estimated breeding values (EBV), it is essential to evaluate a wide variety of polled breeding strategies in terms of long-term selection response and future true genetic relationships by applying simulation techniques. Simulation studies have a long tradition in population genetics to evaluate the effects of evolutionary as well as anthropogenic processes, and have gained additional importance with the rapid development of genomic methods and the increased availability of powerful computer systems [31, 32]. Nonetheless, the availability of specialized software packages using deterministic as well as stochastic approaches that are developed to tackle issues directly targeting animal breeding combined with mating systems is rather limited [33–36]. Deterministic simulations allow equation-based prediction of average genetic gain and average inbreeding level without considering specific individuals. Results from deterministic simulations that addressed selection for the polled trait clearly showed a loss in genetic gain, and steady or decreasing average inbreeding depending on the chosen selection strategy [24]. Inbreeding reduction following selection for polledness was recently confirmed by stochastic simulations [37]. However, short-term inbreeding reduction due to the use of polled sires that are very related between each other, but not so strongly related to the horned populations, will be eroded with high probability in a long-term breeding perspective [22, 38]. Traditionally, for a multiple-trait approach, both deterministic and stochastic simulation techniques require genetic (co)variance components for both traits. Regarding the situation with polledness, only assumptions can be made since results are not available yet.

QMSim [36] is a powerful whole-genome stochastic simulation program that was designed to simulate a wide range of genetic and genomic architectures and population structures, particularly in livestock. Nonetheless, QMSim is limited to the simulation of a single quantitative trait, but includes an interface that can be used for, e.g., the external estimation of breeding values. On the basis of QMSim and with the intention to use the mentioned interface, we developed an R program as an external extension to simulate an additional qualitative polled trait. To our knowledge, there is no stochastic simulation software package available that, simultaneously, simulates a quantitative trait combined with a Mendelian trait (such as the *polled* allele) within the framework of complex dairy cattle breeding programs with multi-trait selection.

Based on the aforementioned simulation technique requirements and the practical need for breeding polled populations, the objectives of this study were: (1) to extend the functionality of the stochastic simulation software QMSim by developing a self-designed R-program for the simulation of an additional qualitative trait; (2) to enable simultaneous selection for the quantitative and qualitative trait using a variety of selection strategies for the polled trait; (3) to evaluate the effects of different selection schemes on the allele frequency of *polled*, genetic gain and inbreeding in a long term perspective.

## Methods

### General programming structure and simulation flow

**Fig. 1:**
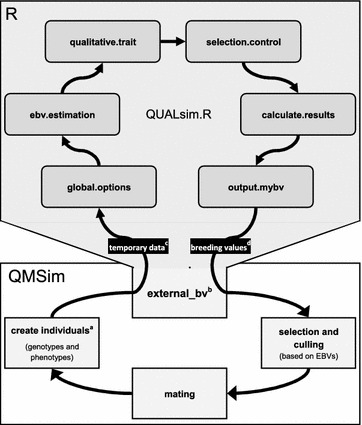
Simulation flow diagram for the combination of QMSim and R_QUALsim. ^a^Start point of the simulation in generation 0. ^b^Option external_bv serves as the interface to connect QMSim and R_QUALsim. ^c^QMSim produces a temporary dataset that is read by R_QUALsim.R after its initiation. ^d^After the successful execution of R_QUALsim, the program creates a file containing the estimated breeding values used for selection in QMSim

The presented simulation framework is a combination of the whole-genome simulation software QMSim [36] for the simulation of a quantitative trait in a dairy cattle population, and an own R algorithm named QUALsim that simulates Mendelian inheritance for a qualitative trait. In the present analysis, QUALsim serves as an extension to simulate polledness in the population initiated by QMSim, and enables simultaneous selection for both traits using various selection strategies. QUALsim and its components were developed and tested using R version 3.2.0 [39]. Programming and testing was performed using the TinnR Editor for the R environment and the associated R package [40]. QUALsim is based on R base functions and functions from community-contributed packages [41, 42]. A detailed technical description of QUALsim and instructions for the usage of QUALsim are provided as a “Technical Note” (see Additional file 1). All the necessary files to run QUALsim with QMSim are in Additional file 2. Figure 1 illustrates the simulation and data flow between QMSim and QUALsim. To date, we have tested QUALsim on Windows and Linux OS systems. The simulation results presented in this study were obtained on a desktop computer system with the following characteristics: operating system (OS) Windows 7 (64 bit); CPU Intel Core i7-4770 3.4 Ghz; 16 GB Ram.

### Population simulation with QMSim

The initial simulation of a dairy cattle population for the quantitative trait was performed by applying QMSim. A quantitative trait (“milk yield”) was simulated as a female sex-limited true polygenic trait with a predefined heritability of 0.3 and a phenotypic variance of 1. True breeding values in generation 0 were set to a mean of 0 with a genetic standard deviation (SD) of 0.54 (i.e. square root of 0.3). Accordingly, no QTL or markers and no historical populations were simulated. The founder generation consisted of 250 male and 50,000 female individuals. From generations 1 to 5, the number of females in the population increased by 12.5 % to reflect the growth of superior breeding lines. After generation 5, the size of the population was kept constant with 250 sires and 75,000 dams. Thirty subsequent generations under selection were simulated. Replacement rates for sires and dams were 50 and 25 % per generation, respectively. Estimated breeding values (EBV) were used as selection and culling criteria for both sires and dams. Selected sires and dams were mated at random. An equal use of selected sires implies 300 offspring per sire and generation. The female reproductive rate was limited to one progeny per female, with an equal probability for either male or female progeny. Each breeding scenario included 20 repetitions.

### Simulation extensions with QUALsim.R

QUALsim.R extends the functionality of QMSim with regard to the subject of our study by processing three main tasks consecutively: (1) EBV estimation for the quantitative trait, (2) simulation of the qualitative trait polledness, and (3) weighting of EBV depending on the simulated polled genotypes or phenotypes.

Breeding values were based on simulated phenotypes from QMSim and estimated by using the external software package DMU [43] (module DMU5). The following animal model was applied:

$${\mathbf{y}} = \mathbf{1}\upmu + {\mathbf{Za}} + {\mathbf{e}},$$

where ${\mathbf{y}}$ is a vector of observations for the quantitative trait, μ is the overall mean of the observations, ${\mathbf{a}}$ is a vector of random additive genetic effects, ${\mathbf{e}}$ is a vector of random residual effects, and ${\mathbf{Z}}$ is the associated incidence matrix for genetic effects.

The polled trait considered as the qualitative trait of interest in this study is assumed to be controlled by a dominant mutant allele at a single locus that determines the polled phenotype. The current status quo for the black-and-white (BWH) polled Holstein population is characterized by high percentages of heterozygous polled individuals, a lower genetic level and higher genetic relatedness, compared to the horned population [21, 22, 24, 26]. Such a realistic genetically-related polled population was initiated by simulating five generations under selection for the quantitative trait. Qualitative trait genotypes and phenotypes were generated by assigning alleles *P* for *polled* and *p* for *horned* at one locus in the progeny of generation 5. Hereafter, the five generations that precede that for which polled genotypes were assigned, will be labeled as generations −5 to −1. Accordingly, generations under selection for both simulated traits are labeled as generations 0 to 25. The polled genotype assignment algorithm implemented in QUALsim allowed for lower breeding values for the quantitative trait, and higher average genetic relationships among polled individuals. The realized polled allele frequency in generation 0 was equal to 0.03 in all simulated breeding scenarios. Average genetic relationships and average true breeding values (TBV) in generation 0 for the polled and horned group reflect the characteristics of the German and international Holstein populations [22, 24] and were similar across scenarios (Table 1). Allele inheritance at the simulated *polled* locus after generation 0 was computed by simulating random combination of parental alleles during mating. Possible evolutionary factors such as recurrent mutations, effects of crossing-over or possible linkage effects in relation to the quantitative trait, were neglected.

**Table 1:** Average pedigree relationship coefficients, inbreeding coefficients and true breeding values ± SD in selected progeny from generation 0 after the assignment of the *polled* allele

	Phenotype			
	Horned		Polled	
	Male	Female	Male	Female
Average relationship coefficient	0.0863 ± 0.0243	0.0338 ± 0.0112	0.1434 ± 0.0504	0.0347 ± 0.0116
Average inbreeding coefficient	0.0233 ± 0.0098	0.0124 ± 0.0049	0.0423 ± 0.0261	0.0130 ± 0.0050
Average true breeding values	2.1111 ± 0.1028	1.5763 ± 0.0743	1.7393 ± 0.1834	1.5273 ± 0.0728

Results were similar across scenarios

Due to the fact that selection and culling of sires and dams in QMSim are strictly based on the EBV, i.e. in our case, estimates from the DMU software package, we developed an alternative approach that allows simultaneous selection for both simulated traits across generations. Our approach weights EBV (allowing a user-defined weighting factor) for the quantitative trait based on individual genotype or phenotype for the qualitative trait. In the present study, initially we used a weighting factor that reflected one genetic SD of the EBV for the quantitative trait (≈weighting factor 0.5 for a quantitative trait with mean = 0 and SD = 1). In the context of the polled breeding scenario evaluations, the weighting factor can be interpreted as an economic weight for the polled trait, i.e., by mimicking a simplified index which includes the polled status of a given individual. We designed two general polled selection strategies.

The first selection strategy GENO weights EBV using the following formula:

$$EBV_{w} = EBV_{quant } + \left( {wf*n_{P} } \right),$$

where $EBV_{quant }$ is the predicted EBV for the quantitative trait of a given individual, *wf* is the chosen weighting factor of 0.5, *n*_*P*_ is the number of *polled* alleles *P* of the individual, and *EBV*_*w*_ is the final weighted EBV given back to QMSim. Hence, the selection strategy GENO refers to marker-assisted selection of polled individuals based on gene test results. GENO implies that all animals are gene-tested at the *polled* locus, and homozygous *polled* individuals are preferably selected.

The second selection strategy PHENO weights EBV using the following formula:

$$EBV_{w} = EBV_{quant } + \left( {wf*PT_{polled} } \right) ,$$

where $EBV_{quant }$ is the predicted EBV for the quantitative trait, *wf* is the chosen weighting factor of 0.5, *PT*_*polled*_ is the binary coded polled phenotype (0 = horned, 1 = polled) of an individual, and *EBV*_*w*_ is the final weighted EBV given back to QMSim. PHENO mimics selection of polled individuals based only on phenotypic information.

While selection strategies that focus on *polled* genotypes (i.e. selection strategy GENO) rely on valid gene tests [19], selection strategies that focus on the polled phenotype (i.e. selection strategy PHENO) might be influenced by different phenotyping errors. In particular, heterozygous polled individuals may develop horn-like skull attachments of variable types, the so-called scurs [11, 44], and at an early calf stage, it can be difficult to phenotypically distinguish between scurs and horns. Such possible phenotyping errors were taken into account when simulating PHENO breeding scenarios. Specifically, we simulated a general phenotyping error rate rather than directly simulating the *scurs* locus as responsible for a second separate qualitative trait for two reasons. First, the precise underlying genetic mechanism of the *scurs* locus is not yet clarified. Second, for both important German cattle breeds Holsteins [22] and Simmental [30], recent evaluations lack detailed information with regard to the allele frequencies of *scurs*. Following our simplified error term strategy, 2 % of all polled progeny in each generation were randomly selected and assigned the horned phenotype, although they were genetically polled.

### Polled breeding scenarios

For the two general selection strategies GENO and PHENO, we designed different sub-selection strategies by imposing EBV weighting to additional constraints, such as sex-specific weighting scenarios. The breeding strategies evaluated here and hereafter referred to as scenarios, comprise a broad range of possible polled selection strategies that include both theoretical scenarios but also scenarios based on practical implementations of commercial farms and breeding organizations. The reference scenario for the comparisons of polled selection scenarios is a base scenario CONTROL, in which there is no targeted selection for the polled trait. Hence, the qualitative polled trait is simulated according to the described methods, but without EBV weighting. Accordingly, selection in scenario CONTROL is strictly based on unweighted EBV for the quantitative trait. Selection scenarios GENO-ALL and PHENO-ALL apply the corresponding general polled selection strategy as explained above in both sexes. Scenarios GENO-M, GENO-F, PHENO-M, PHENO-F are gender-dependent polled selection strategies by weighting EBV only in one sex (M = only among males, F = only among females). In addition, the scenario GENO-M-PHENO-F applies GENO selection among males and PHENO selection among females.

## Results and discussion

### Allele, genotype and phenotype frequencies

**Fig. 2:**
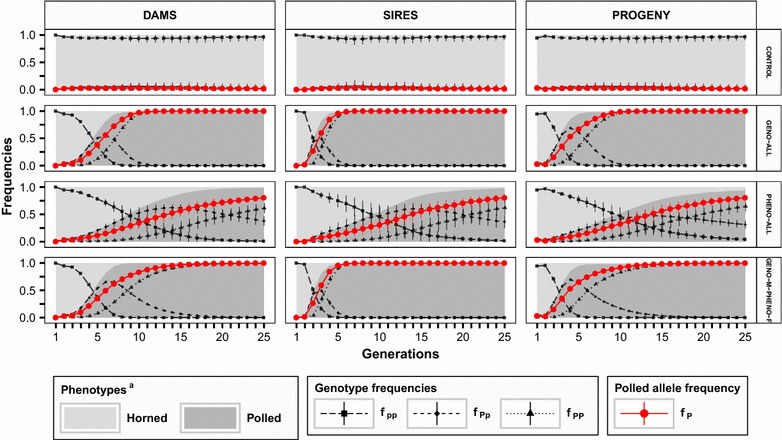
Evolution of genotype, allele and phenotype frequencies across 25 generations in the active population and progeny. Active population = selected individuals. ^a^Sizes of the colored areas are proportional to the percentages of phenotypes in the active population and progeny

The CONTROL scenario, which reflects the traditional breeding and selection strategy applied in black-and-white Holstein cattle, is characterized by a further decrease of the initial allele frequency for *polled* from f_P_ = 0.03 in generation 0 to 0.02 in generation 25 (Fig. 2). In several CONTROL runs, the *polled* allele is even totally eliminated from the active population as reflected by the SD from 20 replicates. The decrease in allele frequency for *polled* with the CONTROL scenario is due to the lower genetic level of polled individuals, as achieved through the initial assignment scheme. Thus, inferior polled individuals in the active population are replaced by superior horned individuals since selection is based strictly on EBV for the quantitative trait regardless of the polled status of an individual.

Both overall polled selection strategies GENO-ALL and PHENO-ALL resulted in phenotypically complete polled active populations. Moreover, application of GENO-ALL resulted in full fixation of the *polled* allele after 18 generations. Coherently, prioritized selection of homozygous individuals was reflected by the genotype frequencies. All polled individuals in scenario GENO-ALL were homozygous polled ongoing from generation 17 (f_PP_ = 1). In contrast, selection for the polled phenotype in scenario PHENO-ALL, regardless of the precise genotypes, retained a significantly larger number of heterozygous individuals in the active population, which resulted in a significantly lower allele frequency for *polled* [p(P) = 0.8022] after 25 generations. Nevertheless, the realized genotype frequencies in scenario PHENO-ALL show that a strict selection on polled individuals based only on phenotype also substantially increased the number of homozygous polled individuals within a time span of 25 generations.

Due to the higher selection intensity, the male selection path was significantly more efficient in breeding polled populations compared to the female selection path (scenarios PHENO-M and GENO-M versus PHENO-F and GENO-F, respectively). The results show that the transition from the low initial allele frequency for *polled* of 0.03 in generation 0 to a high percentage of polled sires and dams in the active population was clearly faster in scenarios GENO-M and PHENO-M compared to female scenarios GENO-F and PHENO-F, respectively (see Additional file 3). Hence, active selection of polled sires accelerated the desired selection effects among dams due to the faster dissemination of *polled* alleles in new born selection candidates.

Remarkably, within only five generations, the sex restricted scenario GENO-M led to polled selection effects among the dams that were equivalent to those with the corresponding scenario with active selection in both sexes (GENO-ALL). In contrast, scenario PHENO-M resulted in a moderate increase in the number of polled dams, but the allele frequency of *polled* among the dams after 25 generations was substantially lower than in the corresponding scenario PHENO-ALL (see Fig. 2; see Additional file 3). Restricting selection for polledness to females (scenarios GENO-F and PHENO-F) only moderately increased the number of heterozygous polled dams, with minor associated selection effects on polled sires. Selection strategies PHENO-F and GENO-F reflect traditional polled selection strategies in Holstein cattle for which polledness is mainly transmitted through the female path [22, 23]. The changes in allele and genotype frequencies for these scenarios were almost identical (see Additional file 3). Therefore, application of polled gene tests for cows kept in commercial herds yields no extra response in the allele frequency of *polled* compared to a selection strategy based on female polled phenotypes.

The sex-dependent combination of both general selection strategies (scenario GENO-M-PHENO-F) led to a phenotypically completely polled active population with an allele frequency of 0.99 for *polled* and 99 % of homozygous polled individuals, as in scenarios GENO-ALL and GENO-M.

### Genetic gain

In the preceding five generations (=generations −5 to −1) before polledness was simulated, the rate of genetic gain was similar and positive in all breeding scenarios (see Additional file 4). For all scenarios, because of the higher selection intensity, the genetic levels of the sires were generally higher than those of the dams. Scenarios with the highest TBV correspond to those with the lowest allele frequencies for *polled* (see also Fig. 2). Accordingly, scenarios with either no or only slight increases in the allele frequency for *polled* (CONTROL, GENO-F, and PHENO-F) displayed higher average TBV than those with significant increases in the allele frequency for *polled* (GENO-ALL, GENO-M, PHENO-ALL and GENO-M-PHENO-F). In the latter scenarios, genetic gain after 25 generations was reduced by 4–2 % compared to the CONTROL scenario (Table 2).

**Table 2:** Mean frequency of the *polled* allele, true breeding values and inbreeding coefficients for 20 replicates ± SD in generation 25

Scenario	*Polled* allele frequency ± SD	True breeding value ± SD	Inbreeding coefficients ± SD
CONTROL	0.0180 ± 0.0354	8.2280 ± 0.1864	0.1249 ± 0.0142
GENO-ALL	1 ± 0	7.9233 ± 0.1810	0.1084 ± 0.0150
GENO-M	0.9983 ± 0.0002	7.9244 ± 0.1635	0.1132 ± 0.0175
GENO-F	0.3228 ± 0.2050	8.0845 ± 0.1854	0.1300 ± 0.0227
PHENO-ALL	0.8022 ± 0.0504	8.0900 ± 0.1127	0.1191 ± 0.0155
PHENO-M	0.5369 ± 0.0782	8.1564 ± 0.1457	0.1274 ± 0.0151
PHENO-F	0.3052 ± 0.1802	8.0813 ± 0.1350	0.1193 ± 0.0165
GENO-M_PHENO-F	0.9986 ± 0.0002	7.9217 ± 0.1434	0.1132 ± 0.0155

Active selection for the polled trait among males (GENO-ALL, GENO-M, PHENO-ALL, PHENO-M and GENO-M-PHENO-F) reduced the average rate of genetic gain per generation in selected sires and dams compared to the CONTROL scenario within 10 generations after the polled allele was assigned. For the later generations 10–25, the rates of average genetic gain for all scenarios were quite constant (see Additional file 4). Final differences in TBV that resulted from reduced rates of genetic gain compared to that of the CONTROL scenario were larger when selection strategies were based on male genotypes (GENO-ALL, GENO-M and GENO-M-PHENO-F) than on male phenotypes (PHENO-ALL and PHENO-M).

The recent evaluations reported by Windig et al. [22] and results for German Red Holstein cattle [24] support the genetic improvement of polled bulls using PHENO strategies. Gaspa et al. [37] applied a moderate PHENO strategy, and found a rather low genetic improvement for polled homo- and heterozygote new-born progeny in a time span of 12 years under conventional BLUP selection and larger losses in rates of genetic gain per year using stochastic simulation. However, the initial parameters that they used, i.e. a rather moderate allele frequency of ~0.10 for *polled,* the significantly lower genetic levels of the polled individuals, the lack of consideration of their relationship level, and the small simulated population, probably explain the small improvements that they observed compared to a more realistic situation. Nonetheless, they identified the potential of further improvements via PHENO strategies when implementing genomic selection [37].

Less overall genetic gain for active selection on polledness is mainly due to long-term selection of male polled selection candidates with lower breeding values for the quantitative trait. Accordingly, a strict preferential selection of homozygous polled sires through higher weighting of their EBV compared to heterozygous polled individuals in scenarios GENO-ALL, GENO-M and GENO-M-PHENO-F retained individuals with lower EBV for the quantitative trait, and excluded genetically-superior horned selection candidates. Polled selection restricted to the female selection path in scenarios GENO-F and PHENO-F showed a comparable effect with increased losses in genetic gain among the active dams compared to the CONTROL dams (see Additional file 4). Thus, practically, polled selection strategies that are restricted to the female pathway may be potentially advantageous for herd performance levels following strict selection of inferior polled cows. Furthermore, allele frequencies for *polled* indicate that active selection for polled males is necessary to achieve sufficient selection responses for polled also in females from a whole population perspective. Accordingly, if only small numbers of polled bulls are available for AI, a moderate selection should be applied to commercial herds using PHENO strategies until more and better polled sires are available from polled breeding programs.

The permanent exclusion of genetically-superior horned selection candidates would not only reduce the genetic potential of the population definitely, but would also unnecessarily decrease the genetic variability of the population. Thus, our simulation results clearly indicate that polled GENO selection strategies should only be applied partially, with moderate intensity and mainly in the male selection pathway by using approaches such as genomic selection [37] and optimum genetic contribution (OGC) theory [22] in future polled breeding programs.

### Inbreeding

Ranking of scenarios according to average inbreeding coefficients generally corresponded to rankings according to TBV, but differences in average inbreeding coefficients among scenarios were quite small (Table 2). The variation of inbreeding coefficients among replicates indicated a substantial impact of individual matings on the actual inbreeding level. A general and similar increase in average inbreeding coefficients as the number of generations increased was observed for all scenarios and for both sexes, with higher levels of inbreeding in bulls than in cows. Average inbreeding rates per generation (ΔF) after generation 0 ranged from 0.312 to 0.576 % (see Additional file 5). Such increases are consistent with recently reported values for the German Holstein and international Holstein populations in the pre-genomic era, these values ranging from 0.44 [45] to 0.95 % [38]. One reason for these slightly lower average inbreeding coefficients in the simulated data could be that the chosen population structure had a relatively small number of active cows compared to the number of active sires, which differs from current practical dairy cattle breeding programs [38]. Nevertheless, we aimed at producing valid inbreeding comparisons across the various polled breeding scenarios, because all scenarios were based on founder populations with the same parameters.

Average inbreeding in the CONTROL scenario showed a consistent linear increase over generations (see Additional file 5). Interestingly, when selecting for the polled trait based on male genotypes (scenarios GENO-ALL, GENO-M and GENO-M-PHENO-F), average inbreeding coefficients after 25 generations were lower than those obtained with the CONTROL scenario. However, the average inbreeding coefficients that were obtained indicated that the lower average inbreeding reached in scenarios GENO-ALL, GENO-M and GENO-M-PHENO-F was mainly due to reduced inbreeding rates in generations 0 to 10. In contrast, in generations 20 to 25, inbreeding rate increased more rapidly, especially among the sires, in scenarios GENO-M and GENO-M-PHENO-F with an assumed impact beyond 25 generations. Average inbreeding coefficients in generation 25 in scenarios PHENO-ALL, PHENO-M, GENO-F and PHENO-F are consistent with those of the CONTROL scenario. In contrast, Gaspa et al. [37] found lower inbreeding rates for a PHENO polled selection strategy using conventional BLUP selection.

Selection based on breeding values from BLUP animal models that combine all the information from relatives contributes to increase the co-selection of related animals with an associated increase in inbreeding [46, 47]. The temporary decrease in average inbreeding as a result of selection for male polled genotypes is partly explained by the selection effects due to the BLUP animal model. Thus, selecting initially only a few individuals and continuously increasing the numbers of polled male and female progeny, decreases average relatedness in the active population by replacing superior and more closely-related horned selection candidates. Such an “alleviation effect” is irrelevant in a long-term perspective with larger proportions of selected polled individuals.

In practice, the group of polled founders (i.e. available polled dams and AI sires already in the population) that could potentially act as donors of the *polled* allele during selection, are highly related [22, 24]. In addition, as shown above, a strict GENO selection strategy cannot be applied in practice because of the implications for genetic gain and performance in the population. Hence, the decrease in inbreeding due to selection based on male polled genotypes (scenarios GENO-ALL, GENO-M and GENO-M-PHENO-F) should not be interpreted as a realistic possibility to reduce inbreeding levels concurrent to polled selection. Instead, the results for the currently practiced PHENO-M selection strategy should be evaluated critically. The high relatedness between potential donors of the *polled* allele could in reality lead to higher inbreeding levels in the long term following an intensified selection for the polled trait [22].

### Important aspects for practical polled breeding

With regard to practical selection decisions, our results strongly suggest that application of GENO selection strategies among males will maximize selection response for polled. The corresponding scenarios GENO-M and GENO-M-PHENO-F resulted in a completely polled active population in a reasonable time span with reduced costs and efforts for genotyping and phenotyping. Scenario GENO-ALL led to a similar result, but the broad genotyping of commercial milking cows at the *polled* locus using the available gene test cannot be carried out in practice due to the current genotyping costs (e.g., 27€ per cow, [19]). As an alternative, imputation of polled genotypes based on marker and pedigree data with low error rates might contribute to broader genotyping activities at an acceptable cost level [24]. From a practical breeding perspective and also considering the costs of genotyping, scenarios GENO-M and GENO-M-PHENO-F seem to be the most efficient strategies to increase the frequency of the *polled* allele in the population. In this context, additional genotyping of females (e.g., as for scenarios GENO-ALL and GENO-M-PHENO-F) resulted only in minor gains regarding the final frequency of the *polled* allele. Commercial herds should focus on balanced selection strategies with regard to the use of available polled AI and elite horned AI sires following traditional selection strategies [23]. Such a strategy requires that potential new born polled progeny be carefully phenotyped, in order to introgress the *polled* allele into the herd. Nevertheless, Segelke et al. [24] suggested an active selection of elite polled females (e.g. potential polled bull dams), which complements the intensive selection among polled males. This suggested strategy contributes to a faster increase of both evaluation criteria i.e. frequency of the *polled* allele and genetic gain among potential polled AI bull selection candidates.

In Holstein AI programs, male polled selection candidates were generally outperformed by horned sires. There are only a few exceptions, e.g. the polled sires Lawn Boy in red Holstein and Mitey P in black-and-white Holstein that disseminated the *polled* allele through the male pathway of selection. Continuous use of only a small number of available polled AI sires has resulted in a population of closely-related polled individuals and in higher inbreeding in the polled subpopulation [22, 24]. The comparison of our results from the simulation with current practical developments indicates that the reported increase in the frequency of the *polled* allele in dairy cattle breeds [22, 30] is consistent with the trend observed in scenarios PHENO-ALL and PHENO-M. Our findings from the GENO scenarios are supported by previously published simulation results [37] and both studies recommend the continued use of gene-tested polled AI sires to achieve high overall frequencies of the *polled* allele within a reasonable time span. The success of the polled AI breeding program in Simmental cattle is exemplarily in this regard [30]. The numerical increase of polled AI sires in black-and-white Holstein in recent years reflects the efforts of the German as well as the international Holstein breeding organizations to broaden the polled sire breeding pool, and to create a basis for structured polled breeding programs [22].

For calf dehorning to be completely abandoned requires 100 % phenotypically-polled new born progeny, which was achieved in scenarios GENO-ALL, GENO-M, GENO-M-PHENO-F within 10 generations, respectively (Fig. 2). However, to maintain a 100 % polled population in the long term requires full fixation of the *polled* allele through selection, which implies a completely homozygous polled active population. In scenario GENO-ALL, all new born progeny are homozygous *polled* ongoing from generation 17. In scenarios GENO-M and GENO-M-PHENO-F, we observed a small number of heterozygous polled progeny up to generation 25. In addition, the results in Fig. 2 clearly illustrate that a selection strategy that includes the genotyped males (GENO-ALL, GENO-M, GENO-M-PHENO-F) is essential to achieve complete polledness in new born progeny. In contrast, selection based on polled phenotypes (PHENO-ALL and PHENO-M) will result in a substantial number of horned progeny still present in generation 25. Specific assortative mating schemes for genotyped polled individuals have the potential to accelerate the breeding process towards polled progeny [25], but in practice, assortative mating schemes are only defined by elite breeders, and with limited applications in commercial herds [48]. Nonetheless, an increasing number of available polled AI bulls with valid gene test results [22, 24, 30] including homozygous *polled* sires, allows commercial farmers to apply assortative polled matings for a faster dissemination of the *polled* allele in their herds.

### Other specific polled breeding applications

We focused on scenario GENO-M-PHENO-F for a further extension of the presented simulation approach aiming at reducing the loss in genetic gain concurrent to the increase in frequency of the *polled* allele (see Fig. 2). For that reason, we changed the weighting factor for GENO selection among males to a lower value of 0.1, while maintaining the high weighting factor of 0.5 for PHENO selection among females. Reducing the male weighting factor (wf-M-0.1) significantly decreased the desired selection response for the polled trait in sires as well as in dams in the first generations (see Table 3; see Additional file 6) compared to wf-M-0.5 (i.e. being the originally simulated GENO-M-PHENO-F scenario). However, the final average frequency of the *polled* allele after 25 generations was equal to 0.93 for dams and 0.98 for sires, which indicated a progressive acceleration of selection response for the qualitative trait. The final overall frequency of the polled phenotype (0.99) also indicated that nearly all the individuals in the active population and new born progeny were polled after 25 generations.

**Table 3:** Further application: mean frequency of the *polled* allele, true breeding values and inbreeding coefficients for 20 replicates ± SD in generation 25

Scenario	*Polled* allele frequency ± SD	True breeding value ± SD	Inbreeding coefficients ± SD
CONTROL	0.0180 ± 0.0354	8.2280 ± 0.1864	0.1249 ± 0.0142
wf-M-0.1	0.9348 ± 0.0353	8.0555 ± 0.1649	0.1210 ± 0.0158
wf-M-0.5	0.9986 ± 0.0002	7.9217 ± 0.1434	0.1132 ± 0.0155

Reducing the weighting factor among males (wf-M-0.1) limited the loss in overall genetic gain for the quantitative trait in sires and dams to 2 % compared to CONTROL (see Table 3; Additional file 7a). Reducing the male weighting factor in scenario wf-M-0.1 resulted in similar average genetic merits for different sire genotypes. In contrast, we found a remaining small deficit in the genetic value of selected polled dams compared to selected horned dams in generation 25. Reducing the weighting factor among males (wf-M-0.1) led to similar inbreeding levels compared to the CONTROL scenario (see Table 3; Additional file 7b).

A fast transition to a completely polled active population and furthermore completely polled progeny is opposed to the preservation of high genetic gain in the quantitative trait. Nonetheless, results from the simulations in which sex-dependent weighting factors were applied, indicate that this decline in genetic gain for the quantitative trait can be limited in combination with significant increases in the proportion of polled individuals. As a compromise, we suggest an approach that takes the described sex-dependent structurally driven effects into account. Hence, it is essential that intensified selection for the polled trait aims at improving the genetic level of polled selection candidates (homozygous as well as heterozygous polled progeny). Such a suggested rather mild selection strategy for the polled trait among male AI candidates is possible with GENO-M and a moderate weighting factor, combined with more intensive selection among females based on polled phenotypes (PHENO-F). This strategy reflects current practical breeding programs that use assortative elite mating schemes and genomic selection, which results in improved EBV for polled Holstein AI bulls [22, 25, 37].

## Conclusions

A fast and lasting dissemination and fixation of the *polled* allele across 25 generations implies a strict selection strategy based on *polled* genotypes. Considering the current characteristics of the available polled AI bulls in most dairy cattle breeds, simulation results indicate that such a strategy is coupled with significant decreases in genetic gain for quantitative performance traits. Selection strategies based only on phenotypic information for the polled trait also led to high frequencies of the *polled* allele, but without its fixation after 25 generations. Such strategies based on phenotype information result in significant increases in the number of heterozygous individuals remaining in the population and in the number of horned progeny born up to the final generations. Therefore, abandoning completely dehorning is not possible when selection is based on polled phenotypes only. The application of polled selection strategies based on gene tests for the male selection pathway combined with moderate weighting of polled genotypes during selection, and a phenotypic polled selection strategy for females using high weighting of polled phenotypes, appears to be the optimal compromise regarding all important evaluation criteria.

## Additional files

10.1186/s12711-016-0228-7 AF1_TechnicalNote_QUALsim.
10.1186/s12711-016-0228-7 AF2_QUALsim.
10.1186/s12711-016-0228-7 Evolution of genotype, allele and phenotype frequencies across 25 generations in the active population and progeny: Additional scenarios. Active population = selected individuals; ^a^sizes of the colored areas are proportional to the percentages of phenotypes in the active population and progeny.
10.1186/s12711-016-0228-7 Average true breeding values (TBV) for active sires and dams across 25 generations. Results for average true breeding values (TBV) of active sires and dams in the CONTROL scenario (labeled as SIRES_CONTROL and DAMS_CONTROL) are included in each plot as a reference.
10.1186/s12711-016-0228-7 Average inbreeding coefficients in the active population across 25 generations. Results for average inbreeding coefficients of active sires and dams in the CONTROL scenario (labelled as SIRES_CONTROL and DAMS_CONTROL) are included in each plot as a reference.
10.1186/s12711-016-0228-7 Further application – Evolution of genotype, allele and phenotype frequencies across 25 generations in the active population for scenario wf-M-0.1 (GENO-M-PHENO-F). Active population = selected individuals. ^a^ the sizes of the colored areas are proportional to the percentages of phenotypes in the active population.
10.1186/s12711-016-0228-7 Further applications (a) Average true breeding values (TBV) for active sires and dams over 25 generations for scenario wf-M-0.1 (GENO-M-PHENO-F) and (b) Average inbreeding coefficients for active sires and dams over 25 generations. Results for average TBV of active sires and dams in the CONTROL scenario (labelled as SIRES_CONTROL and DAMS_CONTROL) are included in each plot as a reference.

## References

[1] Stafford KJ, Mellor DJ (2005). Dehorning and disbudding distress and its alleviation in calves. Vet J.

[2] Stafford KJ, Mellor DJ (2011). Addressing the pain associated with disbudding and dehorning in cattle. Appl Anim Behav Sci.

[3] Morisse JP, Cotte JP, Huonnic D (1995). Effect of dehorning on behaviour and plasma cortisol responses in young calves. Appl Anim Behav Sci.

[4] Faulkner PM, Weary DM (2000). Reducing pain after dehorning in dairy calves. J Dairy Sci.

[5] Heinrich A, Duffield TF, Lissemore KD, Millman ST (2010). The effect of meloxicam on behavior and pain sensitivity of dairy calves following cautery dehorning with a local anesthetic. J Dairy Sci.

[6] Graf B, Senn M (1999). Behavioural and physiological responses of calves to dehorning by heat cauterization with or without local anaesthesia. Appl Anim Behav Sci.

[7] Guatteo R, Levionnois O, Fournier D, Guémené D, Latouche K, Leterrier C (2012). Minimising pain in farm animals: the 3S approach—‘Suppress, Substitute, Soothe’. Animal.

[8] Waiblinger S, Menke C. D.2.3.1. Report on practical recommendations at farm level for keeping horned cattle and on the use of genetic solutions. Alternatives to castration and dehorning (ALCASDE; SANCO/2008/D5/018); 2009.

[9] Menke C, Waiblinger S, Fölsch DW, Wiepkema PR (1999). Social behaviour and injuries of horned cows in loose housing systems. Anim Welf.

[10] Goonewardene LA, Price MA, Liu MF, Berg RT, Erichsen CM (1999). A study of growth and carcass traits in dehorned and polled composite bulls. Can J Anim Sci.

[11] Wiedemar N, Tetens J, Jagannathan V, Menoud A, Neuenschwander S, Bruggmann R (2014). Independent polled mutations leading to complex gene expression differences in cattle. PLoS One.

[12] Tetens J, Wiedemar N, Menoud A, Thaller G, Drögemüller C (2015). Association mapping of the scurs locus in polled Simmental cattle—evidence for genetic heterogeneity. Anim Genet.

[13] Rothammer S, Capitan A, Mullaart E, Seichter D, Russ I (2014). The 80-kb DNA duplication on BTA1 is the only remaining candidate mutation for the polled phenotype of Friesian origin. Genet Sel Evol.

[14] Medugorac I, Seichter D, Graf A, Russ I, Blum H, Göpel KH (2012). Bovine polledness—an Autosomal dominant trait with allelic heterogeneity. PLoS One.

[15] Mariasegaram M, Reverter A, Barris W, Lehnert SA, Dalrymple B, Prayaga K (2010). Transcription profiling provides insights into gene pathways involved in horn and scurs development in cattle. BMC Genomics.

[16] Mariasegaram M, Harrison BE, Bolton JA, Tier B, Henshall JM, Barendse W (2012). Fine-mapping the POLL locus in Brahman cattle yields the diagnostic marker CSAFG29. Anim Genet.

[17] Capitan A, Grohs C, Gautier M, Eggen A (2009). The scurs inheritance: new insights from the French Charolais breed. BMC Genet.

[18] Allais-Bonnet A, Grohs C, Medugorac I, Krebs S, Djari A, Graf A (2013). Novel insights into the bovine polled phenotype and horn ontogenesis in Bovidae. PLoS One.

[19] GeneControl GmbH. 2015. http://www.genecontrol.de/e_hornlosigkeit.html. Accessed 11 Jun 2015.

[20] Schafberg R, Swalve HH (2015). The history of breeding for polled cattle. Livest Sci.

[21] Windig JJ, Eggen A. D.2.2.2. Report on the assessment of breeding strategies in relation to the introduction of the polled gene. ALCASDE: Study on the improved methods for animal-friendly production. In particular on alternatives to the dehorning of cattle. Appendix 23. 2009. http://ec.europa.eu/food/animal/welfare/farm/docs/calves_alcasde_d-2-2-2.pdf.

[22] Windig JJ, Hoving-Bolink RA, Veerkamp RF (2015). Breeding for polledness in Holstein cattle. Livest Sci.

[23] Specht L. Polled Holstein history. 2008. 10.1016/j.livsci.2015.05.017. http://extension.psu.edu/animals/dairy/documents/polled-holsteins-history. Accessed 4 May 2015.

[24] Segelke D, Täubert H, Reinhardt F, Thaller G (2013). Chances and limits of breeding polled cattle Deutsche Holstein. Züchtungskunde.

[25] Spurlock DM, Stock ML, Coetzee JF (2014). The impact of 3 strategies for incorporating polled genetics into a dairy cattle breeding program on the overall herd genetic merit. J Dairy Sci.

[26] Scheper C, Yin T, König S. Inclusion of polled geno- or phenotype into breeding goals: Impact on genetic gain and inbreeding. In: Proceedings of the 66th annual meeting of the European association for animal production, 31 August–4 September 2015, Warsaw; 2015.

[27] Lamminger A, Hamann H, Röhrmoser G, Rosenberger E, Kräusslich H, Distl O (2000). Relationships between polledness and traits used in the breeding objectives for German Fleckvieh. Züchtungskunde.

[28] Goonewardene LA, Pang H, Berg RT, Price MA (1999). A comparison of reproductive and growth traits of horned and polled cattle in three synthetic beef lines. Can J Anim Sci.

[29] Frisch JE, Nishimura H, Cousins KJ, Turner HG (1980). The inheritance and effect on production of polledness in four crossbred lines of beef cattle. Anim Prod.

[30] Götz KU, Luntz B, Robeis J, Edel C, Emmerling R, Buitkamp J (2015). Polled Fleckvieh (Simmental) cattle—current state of the breeding program. Livest Sci.

[31] Hoban S, Bertorelle G, Gaggiotti OE (2011). Computer simulations: tools for population and evolutionary genetics. Nat Rev Genet.

[32] Daetwyler HD, Calus MPL, Pong-Wong R, de Los Campos G, Hickey JM (2013). Genomic prediction in animals and plants: simulation of data, validation, reporting, and benchmarking. Genetics.

[33] Peng B, Chen H, Mechanic LE, Racine B, Clarke J, Clarke L (2013). Genetic simulation resources: a website for the registration and discovery of genetic data simulators. Bioinformatics.

[34] Täubert H, Reinhardt F, Simianer H. ZPLAN+: A new software to evaluate and optimize animal breeding programs. In: Proceedings of the 9th world congress on genetics applied to livestock production, 1–6 August 2010, Leipzig; 2010.

[35] Rutten MJM (2002). SelAction: software to predict selection response and rate of inbreeding in livestock breeding programs. J Hered.

[36] Sargolzaei M, Schenkel FS (2009). QMSim: a large-scale genome simulator for livestock. Bioinformatics.

[37] Gaspa G, Veerkamp RF, Calus MPL, Windig JJ (2015). Assessment of genomic selection for introgression of polledness into Holstein Friesian cattle by simulation. Livest Sci.

[38] Koenig S, Simianer H (2006). Approaches to the management of inbreeding and relationship in the German Holstein dairy cattle population. Livest Sci.

[39] R Core Team. R: a language and environment for statistical computing. 2015. http://www.R-project.org/. Accessed 01 Feb 2015.

[40] Faria JC. Resources of Tinn-R GUI/Editor for R Environment 2012. Ilheus, Bahia, Brasil.

[41] Coster A. Pedigree: pedigree functions. 2012. http://CRAN.Rproject.org/package=pedigree. Accessed 01 Feb 2015.

[42] Wickham Hadley (2011). The split-apply-combine strategy for data analysis. J Stat Softw.

[43] Madsen P, Sørensen P, Su G, Damgaard LH, Thomsen H, Labouriau R. DMU—a package for analyzing multivariate mixed models. In: Proceedings of the 8th world congress on genetics applied to livestock production, 13–18 August, 2006, Belo Horizonte; 2006.

[44] Long CR, Gregory KE (1978). Inheritance of the horned, scurred and polled condition in cattle. J Hered.

[45] Stachowicz K, Sargolzaei M, Miglior F, Schenkel FS (2011). Rates of inbreeding and genetic diversity in Canadian Holstein and Jersey cattle. J Dairy Sci.

[46] Verrier E, Colleau JJ, Foulley JL (1993). Long-term effects of selection based on the animal model BLUP in a finite population. Theor Appl Genet.

[47] Bijma P, Woolliams JA (2000). Prediction of rates of inbreeding in populations selected on best linear unbiased prediction of breeding value. Genetics.

[48] Wensch-Dorendorf M, Yin T, Swalve HH, König S (2011). Optimal strategies for the use of genomic selection in dairy cattle breeding programs. J Dairy Sci.

