# Supplementary material for: Evaluation of breeding strategies for polledness in dairy cattle using a newly developed simulation framework for quantitative and Mendelian traits
Source: Genet Sel Evol. 2016 Jun 29;48:50. doi: 10.1186/s12711-016-0228-7 (PMC4926303; doi:10.1186/s12711-016-0228-7)
Supplement: Supplementary file 1 — 10.1186/s12711-016-0228-7 AF1_TechnicalNote_QUALsim. [file 12711_2016_228_MOESM1_ESM.pdf]

Technical Note for QUALSim (usage in combination with QMSim) June 2016

Author: Carsten Scheper Contact: cscheper@uni-kassel.de

R (Version and used packages)

QUALsim and its components were developed and tested using R version 3.2.0 [39]. Programming and testing was performed using the TinnR Editor for the R environment and the associated R package [40]. QUALsim is mainly based on R base functions. Additional R packages necessary to use QUALsim are "PYLR" and "pedigree" [41,42].

Combined execution of QMSim and QUALsim

The QMSim option 'external_bv' invoking an external solver to estimate breeding values externally serves as an interface to connect QMSim with QUALsim during subsequent generations. QUALsim consists of a main R routine and specific R sub-routines. The main routine QUALsim.R (included in additional file 1, QUALsim.zip) controls the general program flow by executing corresponding R sub-routines pooled in QUALsim_functions.R (included in additional file 1, QUALsim.zip) for i) breeding value estimation, ii) simulation of the qualitative trait, and iii) initiation of selection strategies. The connection between QMSim and QUALsim.R is established through the native Rscript "shell front end" (Rscript is generally installed with R in the standard installation process), which enables the direct execution of R routines from the command line similar to a stand-alone executable. Precisely, if the option 'external_bv Rscript QUALsim.R' is chosen in the QMSim parameter file, QMSim executes Rscript and Rscript then executes QUALsim.R. The simulation en bloc is initialized by operating system (OS) dependent modified QMSim parameter files which contain all required parameters for QMSim as well as additional required parameters for QUALsim to control the simulation of the qualitative trait and the selection strategy (files QUALsim_Windows.txt and QUALsim_Linux.txt included in additional file 1, QUALsim.zip). So far, we have tested QUALsim on Windows as well as Linux OS systems.

The internal structure of QUALsim is modularized including five program elements which are processed consecutively during the simulation of every generation. Utilization of QUALsim in combination with QMSim requires storing of QUALsim.R, QUALsim_functions.R and the modified parameter file suitable for the used operating system (QUALsim_Windows.txt or QUALsim_Linux.txt) directly in the QMSim working directory. Additionally, it is mandatory that the R binary directory is set in the systems PATH variable, i.e., to enable execution of RScript directly from the QMSim work folder. The simulation process within QUALsim includes the following modules in chronological order (further specific technical information regarding the internal programming structure are included in the commented QUALsim program files in additional file 1).

Module 1: global.options

The "global options" element processes the parameter settings from the parameter file by reading in all relevant parameters as temporary variables, being accessible during the simulation process in the R environment. Necessary parameters especially include the specification of the assignment scheme for the qualitative trait, of the selection strategy, and of the EBV estimation software package. During the first simulation run in the first repetition when creating the founder generation, settings in "global.options" allow to create specific subdirectories for organized storage of QUALsim output files during simulation.

Module 2: ebv.estimation

The "ebv.estimation" module estimates breeding values based on the QMSim-phenotypes by utilizing external software packages. So far we implemented sub-routines to use two software packages for breeding value estimation: DMU [4] (Windows and Linux using module DMU5) and PEST [6] (Linux only). It is imperative to set the executables as PATH variables. Otherwise, the user is requested to copy the respective executables into the QMSim working directory. A parameter file for DMU as used in this study is provided as a supplemental file (QUALsim_DMU5 included in additional file 1 QUALsim.zip, suitable for Windows and Linux).
Breeding values are predicted using a simple animal model:

$$\mathbf{y} = \boldsymbol{\mu} + \mathbf{Za} + \mathbf{e}$$

where **y** = vector of observations for the quantitative trait, **μ** is the overall mean of the observations, **a** = vector of random additive genetic effects, **e** = vector of random residual effects, and **Z** is the associated incidence matrix for genetic effects.
Technically, "ebv.estimation" uses a temporary QMSim output file "data.tmp" as basis for the preparation of separated pedigree and phenotype files as required by the external software packages. Subsequent to the data preparation the breeding value estimation software is executed by QUALsim using the R system() function as a shell front end. Solutions for random additive genetic effects (i.e. animal EBVs) are optinally stored for further processing and evaluation.

Selectable Options:

ebv.estimation:

DMU

Breeding values are estimated using DMU (module DMU5). EBV estimation using DMU works on Windows as well as Linux systems. Please make sure that the provided DMU parameter file (QUALsim_DMU5.DIR) is stored in the QMSim working folder.

PEST

Breeding values are estimated using PEST. EBV estimation using PEST works on Linux systems only. Please make sure that the provided PEST parameter file (QUALsim_PEST.txt) is stored in the QMSim working folder.

save.EBVs

if set to **yes**, EBVs for all generations are stored in a data file (df_ebv) at the end of a repetition run for traceability and manual calculation of breeding value estimation accuracy. Have in mind that with big populations and high generation numbers the size of the data file can reach high values beyond 10GB. Limited disk space will lead to simulation abort in case of limited disk space.

If no value is set in the parameter file no EBVs will get stored by default

Module 3: qualitative.trait

Simulation of an additional qualitative trait and Mendelian inheritance is the key component of QUALsim. The simulated qualitative trait is initially generated by assigning two alleles (i.e. one dominant allele coded with 1, one recessive allele coded with 0) at one locus in the selected generation, determining the phenotype of a respective individual (i.e. individuals with at least 1 dominant allele receive a phenotype coded with 1, individuals with no dominant allele receive a phenotype coded with 0). Allele inheritance in following generations is then computed by simulating recombination of parental alleles, mimicking the natural process of recombination of parental alleles during mating. Possible evolutionary factors such as recurrent mutations or effects of crossing over events on the specific qualitative trait single locus, are not considered. In the consecutive inheritance, recombination of polled alleles in progeny is furthermore completely independent from the quantitative trait genetic value disregarding possible linkage effects.
Implemented options for allele assignment enable to simulate a broad and flexible range of trait and breeding scenarios. Initial allele assignment can either be completely randomized, and/or based on gender, relationships and genetic and phenotypic values, always in relation to pre-defined allele frequencies or genotype counts. For technical details please see the qualitative trait assignment sub-routines in the supplemental program file QUALsim_functions.R (included in additional file 1, QUALsim.zip). Further modifications and extensions are easily possible by defining own specific sub-routines or adapting the already included sub-routines using the provided framework in the supplemental program file QUALsim_functions.R (included in additional file 1, QUALsim.zip).

Selectable Options:

qual.assign.gen

Defines the generation number in which (qualitative trait) alleles are assigned utilizing the chosen assignment mode and scheme. Qualitative trait alleles are assigned in progeny from the specified generation. Assignment of qualitative trait alleles in generations after the initial founder generation of the simulation (Gen 0) enables assignment options that take relationships between simulated individuals into account (see option low_tbv_high_R) to depict specific situations regarding relationship or inbreeding coefficients. In the corresponding study we used assignment.scheme

option low_tbv_high_R to mimic lower breeding values and higher relatedness in polled animals after the assignment of qualitative trait genotypes in progeny from generation 5.

assignment.mode:

defined_scenario

Assignment of qualitative trait alleles is based on a genotype count scheme. Hence, the number of homo- and heterozygous male and female individuals for the qualitative trait alleles is predefined using a text file named **GT_count_scenario** (an example file used in the corresponding study is included in additional file 1, QUALsim.zip) …

allele_frequency

Assignment of qualitative trait alleles is based on a user-defined allele frequency for the dominant allele of the quantitative trait. Based on the given allele frequency and the simulated population size, the number of dominant alleles to be assigned is calculated and assigned using the selected assignment.scheme. If only one value is defined, a population wide allele frequency is assumed and assignment is performed among all individuals at once. If two values are defined, the first value specifies the allele frequency among males while the second value specifies the allele frequency among females allowing for sex-specific allele frequencies.

assignment.scheme:

rnd

Qualitative trait alleles (assignment.mode allele_frequency) or genotypes (assignment.mode defined_scenario) are assigned completely randomized among progeny in the specified generation selected for the qualitative trait assignment (see option qual.assign.gen)

low_tbv:

Progeny in the specified generation selected for the qualitative trait assignment (see option qual.assign.gen) is divided into four different groups according to quartiles of the distribution of true breeding values (TBVs) for the quantitative trait.

If assignment.mode defined_scenario is selected the defined number of homozygote genotypes for the qualitative trait (i.e homozygous polled animals in the corresponding study) is randomly assigned within the group of individuals with TBVs in the fourth and third quartile while the number of heterozygote genotypes is randomly assigned within the group of individuals with TBVs in the second and third quartile.

If assignment.mode allele_frequency is selected the respective number of alleles for the qualitative trait (i.e in the corresponding study polled alleles) is randomly assigned within the group of individuals with TBVs in the fourth, third and second quartile.

low_tbv_high_R

In a first step influential sires and grandsires with progeny in the specified generation selected for the qualitative trait assignment (see option qual.assign.gen) are identified and ranked primary based on average relatedness to each other and secondary on TBVs. In a second step sire combinations are consecutively selected from the ranked group of influential sires until the number of progeny descending from those selected sire combinations

sufficiently exceeds the defined number of male and female individuals for the assignment of qualitative trait alleles (assignment.mode allele_frequency) or genotypes (assignment.mode defined_scenario). Progeny from the selected sire group in the selected assignment generation (see qual.assign.gen) serve as potential candidates for the assignment.

Males and females are selected for the assignment of qualitative trait alleles or genotypes by iteratively (500 iterations) sampling the selected amounts of males and females from assignment candidates and comparing their average relatedness. Basis is the pedigree relationship matrix for all potential assignment candidates containing relationship coefficients. Alleles or genotypes are finally randomly assigned in the selected group of males and females showing the highest relatedness of all iterations.

phenotyping.error.rate

Although qualitative traits follow dominant/recessive or intermediate inheritance patterns with clearly distinct phenotypes, phenotyping errors may occur in practice due to different reasons. Lack of phenotype knowledge among handling personnel paired with limited time-resources likely leads to potentially significant amounts of wrongly phenotyped individuals. Additionally trait interactions could further complicate sufficient phenotyping results. In case of polledness, the qualitative trait of concern in the corresponding study, the occurrence of scurs in heterozygous polled individuals might lead to some polled animals wrongly phenotyped as horned.

The option **phenotyping.error.rate** enables the consideration of phenotyping error rates and their effect on selection. A value for phenotyping error rates should be given as a ratio between 0 and 1 (for example for 2% phenotyping error rate select 0.2). If a value for **phenotyping.error.rate** is set, a corresponding amount of individuals showing the dominant phenotype (i.e. in case of the corresponding study polled animals) is randomly selected and receives the recessive genotype (i.e. animals will be wrongly marked as horned even though they carry polled alleles).

Module 4: selection.control

Due to the fact that selection and culling of sires and dams in QMSim is based on EBVs, i.e. in our case estimates from DMU or PEST, we developed an alternative approach allowing simultaneous selection for both simulated traits during generations. Our approach utilizes weighted EBVs for the quantitative trait in dependency of genotypes or phenotypes for qualitative trait. EBV weighting is implemented in the module "selection.control", and allowing a user defined weighting factor. In the present study, we initially used a weighting factor reflecting one genetic SD of the EBV for the quantitative trait (≈ 0.5 for a quantitative trait with mean = 0 and SD = 1). In the context of the polled breeding evaluations, the weighting factor can be interpreted as an economic weight for the polled trait, i.e. to simulate a simplified index breeding value that includes the polled status of a given individual. We designed two general polled selection strategies:

The first selection strategy GENO weights EBVs using the following formula:

$$EBV_w = EBV_{quant} + (wf * n_P)$$

where EBV_{quant} is the predicted EBV for the quantitative trait, wf is the chosen weighting factor of 0.5, n_P are the number of polled alleles P of an individual, and EBV_w is the final weighted EBV given back to QMSim. Hence, the selection strategy GENO mimics marker-assisted selection of polled individuals based on gene-test results. GENO implies that all animals are gene-tested at the polled locus, and homozygote polled individuals are preferably selected. The second selection strategy PHENO weights EBVs using the following formula:

$$EBV_w = EBV_{quant} + (wf * PT_{polled})$$

where EBV_{quant} is the predicted EBVs for the quantitative trait, wf is the chosen weighting factor of 0.5, PT_{polled} is the binary coded polled phenotype (0 = horned, 1 = polled) of an individual, and EBV_w is the final weighted EBV given back to QMSim. PHENO mimics selection of polled individuals based only on phenotypic information. Within the two general selection strategies, we designed a broad range of different sub-selection strategies for the polled trait by imposing EBV weighting to additional conditions, such as sex specific weighting scenarios. The developed weighting approach provides a framework for a broad diversity of possible selection strategies up to the selection of single individuals, or even the implementation of a full selection index approach combined with optimum genetic contribution (OGC) theory.

Selectable Options:

weighting.factor:

The selected weighting factor is multiplied with the number of dominant qualitative trait alleles (selection.scheme GENO) or dominant qualitative trait phenotype (selection.scheme PHENO) and added to the initially estimated EBV to enable selection for the qualitative trait. The chosen factor should correspond to the defined value for the phenotypic variance for the quantitative trait to ensure sufficient selection effects. If a single value is given, the weigthing factor is applied population wide. If two values are given, the first value defines the weighting factor applied in males, the second value defines the weighting factor in females.

selection.scheme:

CONTROL

Serves as a basis scenario for comparisons with selection scenarios applying selection for the qualitative trait. Selection.scheme CONTROL is strictly based on unweighted EBVs for the quantitative trait without a targeted selection for the simulated qualitative trait.

GENO-ALL

EBVs of both males and females are weighted according to general selection strategy GENO. Homozygous animals carrying two dominant alleles are preferably selected.

GENO-M

EBVs only of males are weighted according to general selection strategy GENO. Male homozygous animals carrying two dominant alleles are preferably selected.

GENO-F

EBVs only of females are weighted according to general selection strategy GENO. Female homozygous animals carrying two dominant alleles are preferably selected.

PHENO-ALL

EBVs of both males and females are weighted according to general selection strategy PHENO. Animals carrying at least one dominant allele are preferably selected.

PHENO-M

EBVs only of males are weighted according to general selection strategy PHENO. Males carrying at least one dominant allele are preferably selected.

PHENO-F

EBVs only of females are weighted according to general selection strategy PHENO. Females carrying at least one dominant allele are preferably selected.

GENO-M-PHENO-F

EBVs of males are weighted according to general selection strategy GENO. Male homozygous animals carrying two dominant alleles are preferably selected.

EBVs of females are weighted according to general selection strategy PHENO. Females carrying at least one dominant allele are preferably selected.

Module 5: output.mybv

In order to continue the simulation of subsequent generations in QMSim, QMSim requires a data file 'my_bv.txt' containing ID and EBV of animals in a specific format. The file 'my_bv.txt' includes two columns and a header as described in the QMSim manual [7] on page 20. The module "output.mybv" creates this file based on the stored results from the modules "ebv.estimation" and "selection.control". Subsequently, processing of QUALsim.R and therefore also of Rscript is terminated. QMSim then searches and reads my_bv.txt and carries on with the simulation.

In addition relevant results for the parameters: quantitative trait true breeding values, qualitative trait allele and genotype frequencies and inbreeding coefficients are calculated in the last generation for a respective repetition. Final average results over repetitions are calculated in the last generation of the last repetition according to the user defined number of generations and repetitions.

Simulation package: Further possible applications

The simulation of a monogenic Mendelian trait using QUALsim in combination with QMSim is not limited to the inheritance of polledness as in the corresponding study. Thus, any monogenic Mendelian trait of relevance can be simulated to study the effects of simultaneous selection for a Mendelian trait and the quantitative "QMSim trait". Simulation of multiple Mendelian traits and corresponding multi-trait selection strategies will be achieved by extending the provided QUALsim program files. The programming framework also considers adaption to similar issues in other species (e.g. swine, poultry etc.) characterized by different breeding structures with a strong focus on nucleus breeding schemes. Such an approach allows monitoring of allele frequency development, inbreeding and genetic gain in different sub-populations, i.e. production unit and nucleus by combining the functionality of QMSim and QUALsim.R. The presented programming approach allows to depict a broad range of specific breeding strategies (e.g. considering of semen sexing, embryo transfer, or

gene introgression), and further specific selection and breeding schemes. Furthermore, QMSim allows to simulate different genome architectures regarding chromosomes, QTL and marker numbers and effects. Marker and QTL data are temporarily written out after every generation similar to TBVs and phenotypes as used in the presented approach. Therefore, the simulation of Mendelian traits using QUALsim for different genome architectures (i.e. marker and QTL data) is possible by adapting the provided assignment sub-routines in QUALsim_functions.R (included in additional file 1)
