# Supplementary figures and images for: Evaluation of breeding strategies for polledness in dairy cattle using a newly developed simulation framework for quantitative and Mendelian traits
Source: Genet Sel Evol. 2016 Jun 29;48:50. doi: 10.1186/s12711-016-0228-7 (PMC4926303; doi:10.1186/s12711-016-0228-7)

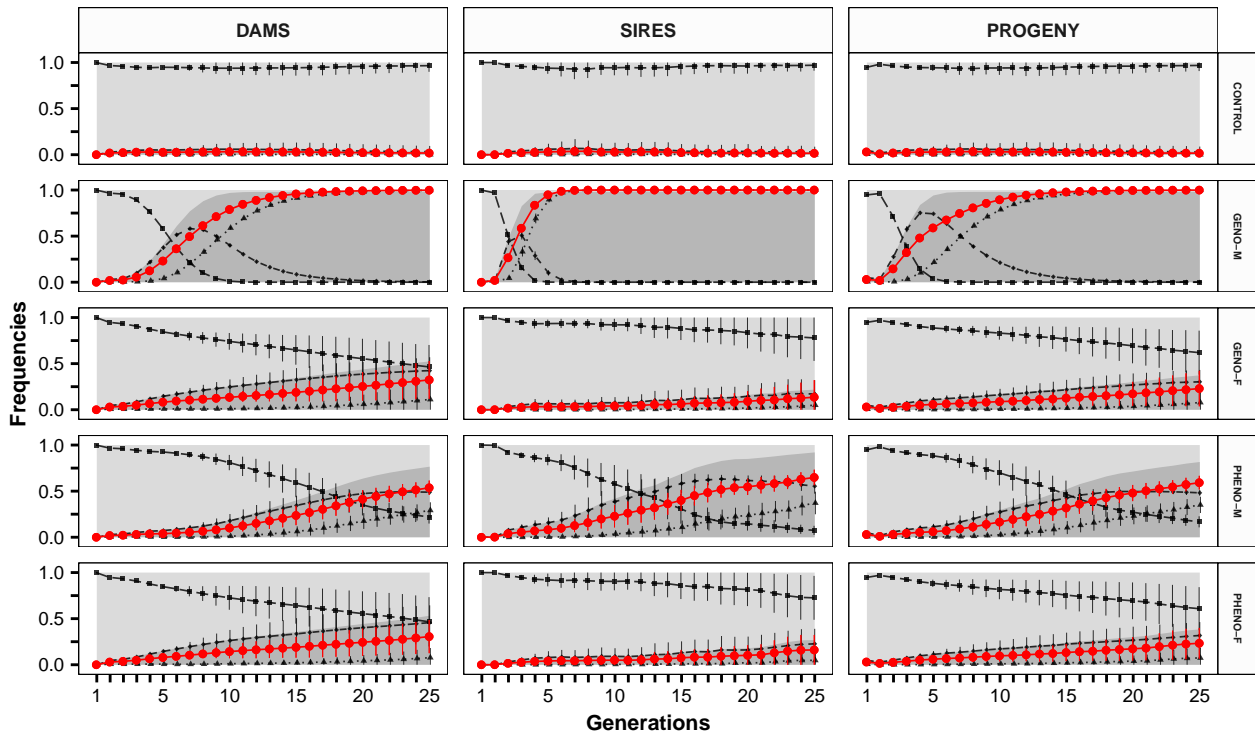

Phenotypes<sup>a</sup>

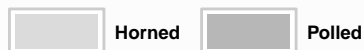

Genotype frequencies

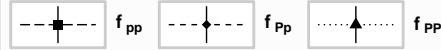

Polled allele frequency

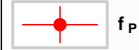

Supplement: Supplementary file 3 — 10.1186/s12711-016-0228-7 Evolution of genotype, allele and phenotype frequencies across 25 generations in the active population and progeny: Additional scenarios. Active population = selected individuals; asizes of the colored areas are proportional to the percentages of phenotypes in the active population and progeny. [file 12711_2016_228_MOESM3_ESM.pdf]

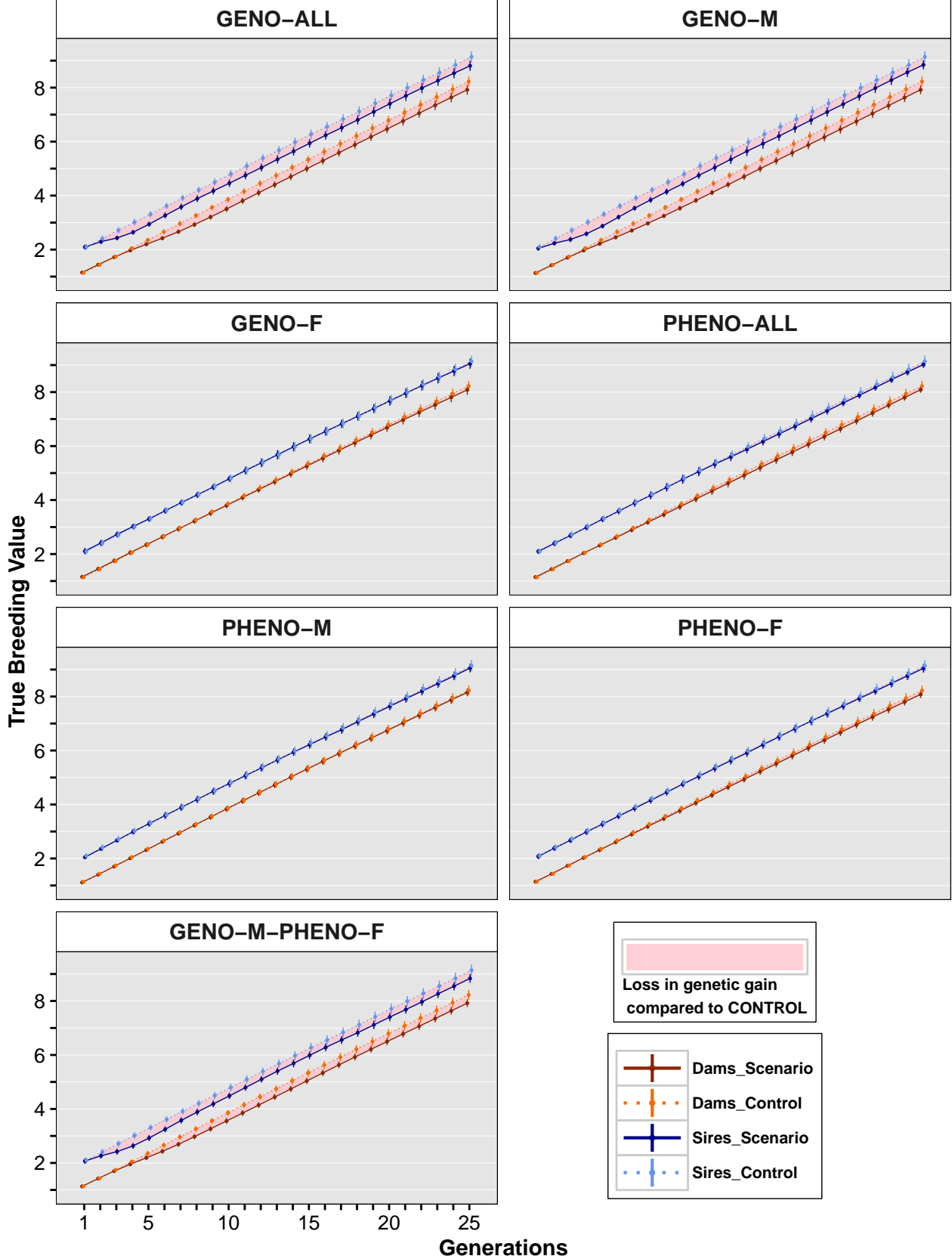

Supplement: Supplementary file 4 — 10.1186/s12711-016-0228-7 Average true breeding values (TBV) for active sires and dams across 25 generations. Results for average true breeding values (TBV) of active sires and dams in the CONTROL scenario (labeled as SIRES_CONTROL and DAMS_CONTROL) are included in each plot as a reference. [file 12711_2016_228_MOESM4_ESM.pdf]

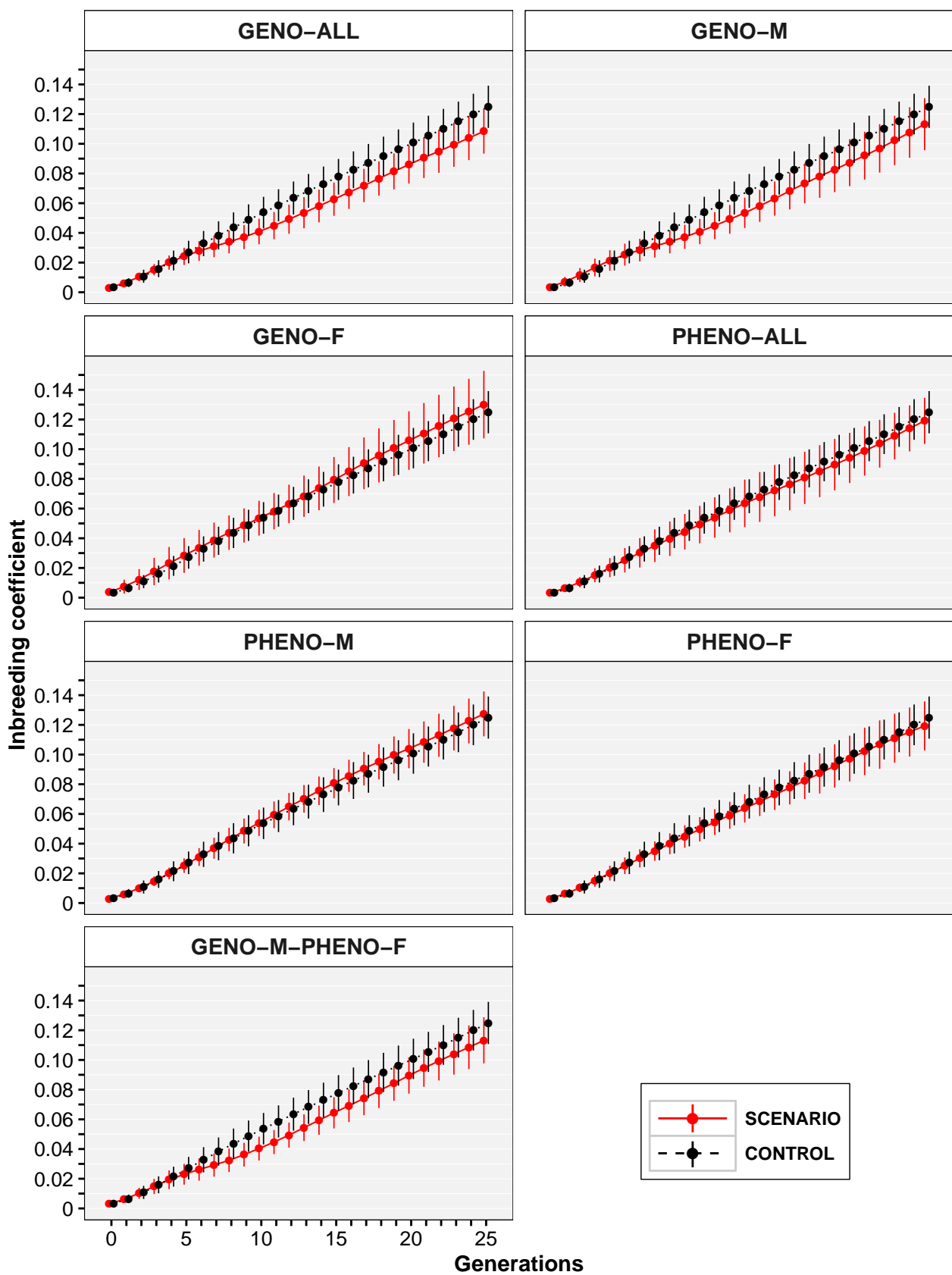

Supplement: Supplementary file 5 — 10.1186/s12711-016-0228-7 Average inbreeding coefficients in the active population across 25 generations. Results for average inbreeding coefficients of active sires and dams in the CONTROL scenario (labelled as SIRES_CONTROL and DAMS_CONTROL) are included in each plot as a reference. [file 12711_2016_228_MOESM5_ESM.pdf]

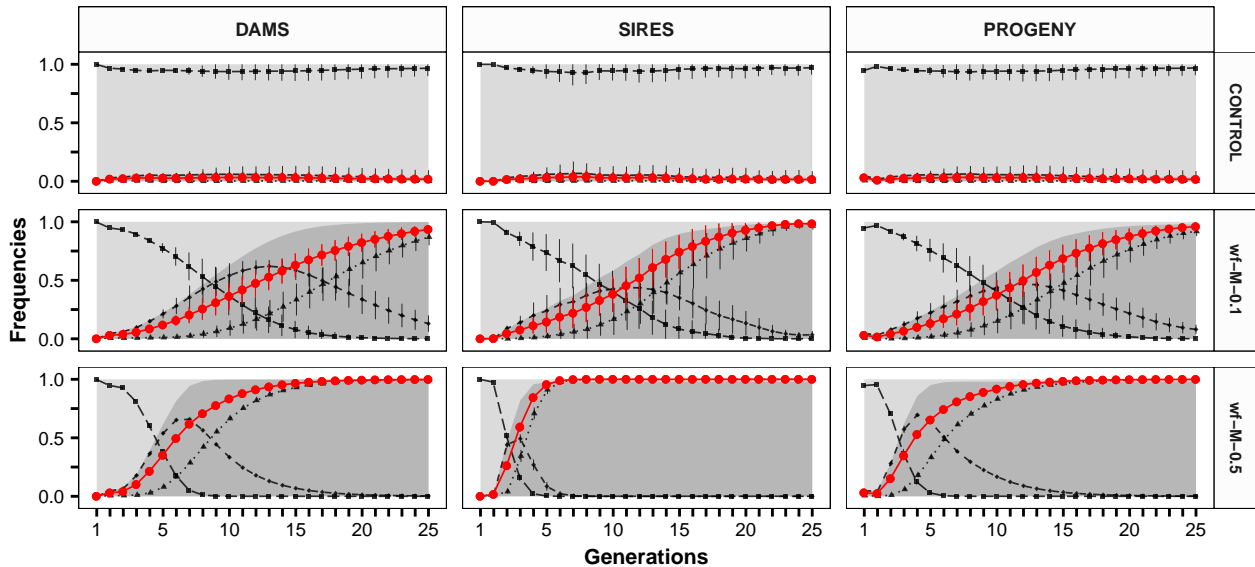

Supplement: Supplementary file 6 — 10.1186/s12711-016-0228-7 Further application – Evolution of genotype, allele and phenotype frequencies across 25 generations in the active population for scenario wf-M-0.1 (GENO-M-PHENO-F). Active population = selected individuals. a the sizes of the colored areas are proportional to the percentages of phenotypes in the active population. [file 12711_2016_228_MOESM6_ESM.pdf]

**a**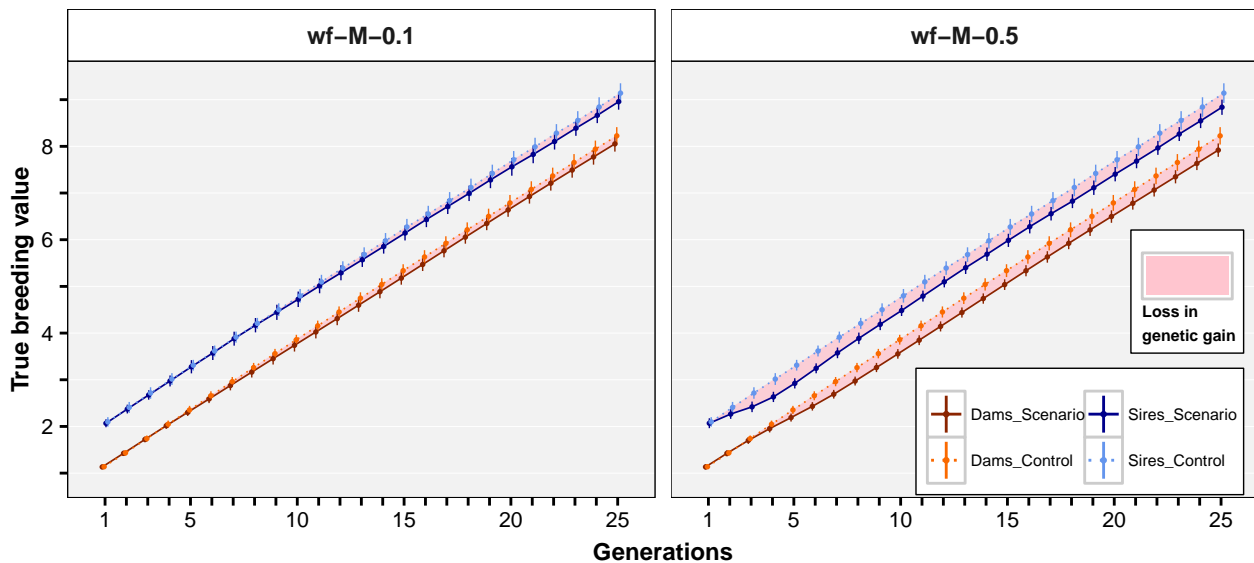**b**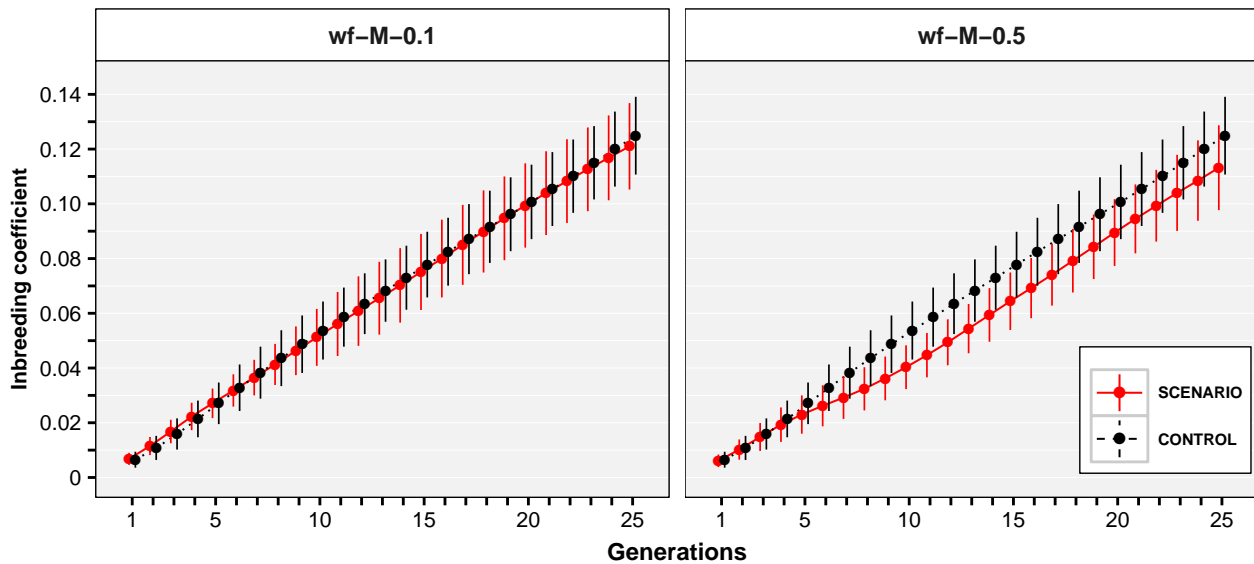

Supplement: Supplementary file 7 — 10.1186/s12711-016-0228-7 Further applications (a) Average true breeding values (TBV) for active sires and dams over 25 generations for scenario wf-M-0.1 (GENO-M-PHENO-F) and (b) Average inbreeding coefficients for active sires and dams over 25 generations. Results for average TBV of active sires and dams in the CONTROL scenario (labelled as SIRES_CONTROL and DAMS_CONTROL) are included in each plot as a reference. [file 12711_2016_228_MOESM7_ESM.pdf]
